# Artificial intelligence in medicine: from molecular data analysis to clinical decision-making

**DOI:** 10.3325/cmj.2026.67.135

**Published:** 2026-06

**Authors:** Dragan Primorac, Thomas C. Südhof

**Affiliations:** 1 St. Catherine Specialty Hospital, Zagreb, Croatia; 2 International Center for Applied Biological Research, Zagreb, Croatia; 3 School of Medicine, Josip Juraj Strossmayer University of Osijek, Osijek, Croatia; 4 Faculty of Dental Medicine and Health, Josip Juraj Strossmayer University of Osijek, Osijek, Croatia; 5 Eberly College of Science, The Pennsylvania State University, State College, PA, USA; 6 School of Medicine, University of Split, Split, Croatia; 7 The Henry C. Lee College of Criminal Justice and Forensic Sciences, University of New Haven, New Haven, CT, USA; 8 Sana Kliniken Oberfranken, Coburg, Germany; 9 School of Medicine, University of Rijeka, Rijeka, Croatia; 10 School of Medicine, University of Pittsburgh, Pittsburgh, PA, USA; 11 National Forensic Sciences University, Gandhinagar, India; *dragan.primorac@svkatarina.hr*; 12 Stanford University, Stanford, CA, USA

Artificial intelligence (AI) is no longer a futuristic concept confined to computational laboratories but is increasingly embedded in daily clinical workflows and research practices. From diagnostic imaging and pathology to genomics and population health, AI systems are redefining how data are interpreted and translated into clinical action and are used in biomedical research. In diagnostic imaging and digital pathology, AI-driven pattern-recognition systems now achieve or even exceed expert-level performance in identifying radiographic, histologic, and cytologic abnormalities. The exponential growth of biomedical data, driven by whole genome sequencing (WGS), high-throughput omics technologies, wearable sensors, and digital health platforms, has surpassed the analytical capacity of traditional data analysis approaches. Large data sets are currently routinely generated in preclinical and clinical studies, rendering AI indispensable, for example, for interpreting genomic sequencing results or clinical trial designs. AI, particularly machine learning (ML) and deep learning (DL), provides the computational framework necessary to integrate the many new multidimensional data sets into biomedically relevant conclusions and clinically meaningful insights.

Modern medicine is undergoing a profound paradigm shift – from reactive, symptom-driven interventions to anticipatory, predictive, and preventive care. This shift is facilitated by AI’s ability to identify subtle and robust patterns across genomic, molecular, imaging, and clinical variables. When multi-omic signatures are fused with phenotypic, environmental, and longitudinal electronic health record (EHR) data, AI converts static clinical snapshots into dynamic, probabilistic disease-trajectory models. These models not only refine diagnosis and prognosis but also stratify risk, personalize therapy selection, and forecast treatment responses with unprecedented granularity. In this sense, AI serves as the integrative backbone of precision medicine, connecting molecular biology with real-time clinical decision-making (1) (Figure 1).

**Figure 1:**
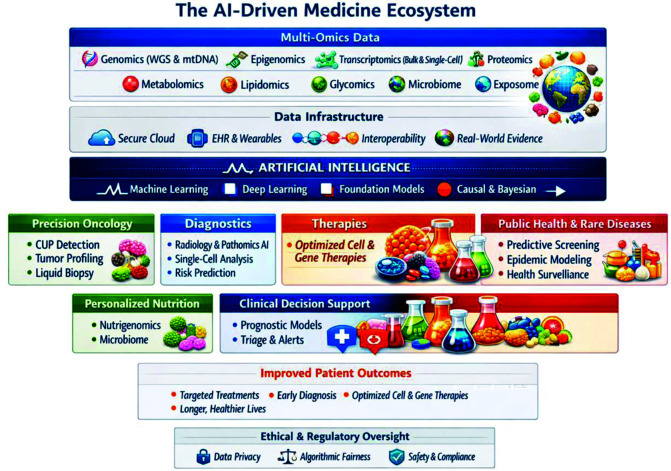
Overview of artificial intelligence (AI)-driven health care, including multi-omics data, artificial intelligence processing pipelines, and translation into clinical practice. WGS – whole genome sequencing; EHR – electronic health records; CUP – cancer of unknown primary.

Similarly, AI is able to identify patterns in otherwise inaccessible data sets, such as epidemiological data coupled to genomic sequencing results on large populations. For example, the UK biobank contains health information from roughly half a million volunteers, including genetic data, medical imaging, lifestyle surveys, and long-term clinical records (2). When these data streams are combined, they form a landscape so intricate that only ML models can realistically navigate it. By using the UK Biobank data, AI models can scan millions of genetic variants alongside decades of health outcomes. For instance, an ML system might detect that a combination of rare variants – each individually insignificant – collectively increases the likelihood of developing a metabolic disorder. These multi-variant interactions are nearly impossible to spot with classical statistical tools because the relationships are nonlinear and buried in noise.

## AI-powered analysis of whole genome sequencing data

Whole genome sequencing (WGS) represents one of the most transformative innovative technologies in contemporary medicine. In contrast to whole exome sequencing (WES), which captures only ~ 1%-2% of the genome, WGS interrogates both coding and non-coding regions, structural variants, copy number variations, mitochondrial DNA, repetitive elements, and regulatory sequences that modulate gene expression. However, each sequenced genome uncovers millions of variants, most of which are benign or of uncertain significance. As a result, the central bottleneck in WGS is no longer data acquisition but the scientific interpretation and clinical contextualization of these vast variant data sets. Robust implementation of WGS in routine care, therefore, depends on advanced computational pipelines capable of distinguishing clinically meaningful variants from background genomic noise. AI significantly enhances this interpretive process. ML algorithms integrate diverse annotation layers – evolutionary conservation metrics, functional prediction scores, transcriptomic and epigenomic data sets, and phenotype-genotype correlations – to identify variants with greater biological significance. Deep neural networks trained on curated variant data sets can infer pathogenicity with increasing precision, particularly for rare variants, non-coding regulatory mutations, and variants with subtle functional effects that evade traditional rule-based classification systems (3).

Moreover, AI facilitates the calculation of polygenic risk scores (PRS), integrating thousands of genetic variants into a single predictive metric. These scores can estimate individual susceptibility to complex diseases such as cardiovascular disease, diabetes, neurodegenerative diseases, and cancers. When combined with environmental exposures and lifestyle data, AI-driven PRS models support early risk stratification and personalized prevention strategies. In oncology, AI-enhanced WGS analysis allows the identification of mutational signatures associated with specific carcinogenic processes, such as DNA repair deficiencies or environmental exposures. These signatures not only improve diagnostic classification but also guide targeted therapy selection and immunotherapy eligibility (4).

## Generative AI-guided biology and drug design

Generative AI is emerging as a powerful tool in biological research and therapeutic innovation because it can discover complex patterns across multi-omics data sets – spanning genomics, transcriptomics, proteomics, metabolomics, and epigenomic profiles – together with protein structures, chemical libraries, and regulatory network architectures. By capturing relationships within these high-dimensional data layers, generative models can design entirely new small molecules, interpret protein sequences, or define regulatory elements that exhibit specific functional or biophysical properties. This capability streamlines the earliest phases of drug discovery by focusing experimental efforts on candidates with the highest likelihood of success.

In medicinal chemistry, generative AI algorithms can refine molecular structures to improve the binding affinity, target selectivity, solubility, and metabolic properties of small molecules. In protein engineering, generative AI can design novel enzymes, therapeutic peptides, or antibody variants with enhanced stability or activity. Integrating multi-omics information allows generative AI models to anticipate how proposed molecules may influence disease-relevant pathways or interact with cellular states derived from patient data. Although these computational approaches can dramatically shorten discovery timelines, every AI-generated candidate still requires rigorous laboratory and preclinical testing to verify biological function, safety, and translational potential.

## AI-driven precision oncology

Cancer exhibits extensive molecular heterogeneity, manifested both between patients (inter-tumor heterogeneity) and within individual tumors (intra-tumor heterogeneity). This heterogeneity encompasses variations in genetic, epigenetic, transcriptional, proteomic, metabolic, and phenotypic features, all of which influence tumor evolution, therapeutic response, and disease outcome. Comprehensive molecular tumor profiling integrating genomics, transcriptomics, epigenomics, and proteomics enables a deeper characterization of oncogenic drivers, tumor subtypes, and resistance mechanisms, thereby informing precision oncology and therapeutic decision-making (5,6).

Radiogenomics represents a particularly promising intersection of AI and oncology. By combining imaging data with molecular tumor profiles, AI models can infer genetic alterations from radiological features. This non-invasive approach may reduce the need for repeated biopsies and enable real-time monitoring of tumor evolution. Importantly, AI-driven oncology platforms are increasingly used in molecular tumor boards, where genomic findings are interpreted within a clinical context. Decision-support systems integrate variant databases, clinical trial repositories, and evidence-based guidelines, assisting clinicians in selecting personalized therapeutic strategies (1,5).

For example, cancers of unknown primary (CUP) remain one of the most challenging oncologic diagnoses. Patients present with metastatic disease, yet conventional imaging and histopathological evaluations fail to identify the primary tumor site. Empirical chemotherapy often yields limited efficacy and poor outcomes. AI offers a powerful solution to this diagnostic dilemma. ML models trained on large data sets of tumor transcriptomic and epigenomic profiles can predict the tissue of origin based on molecular signatures. These classifiers analyze gene expression patterns, methylation profiles, and mutational landscapes to assign a probability score for potential primary sites. Such AI-based CUP identification tools enable targeted therapies aligned with a tumor’s origin rather than relying on non-specific regimens. Beyond diagnosis, AI models can predict likely treatment responses and survival, facilitating risk-adapted management. As these algorithms continue to improve, they may significantly enhance prognosis in a historically underserved patient population (7,8).

As another example, liquid biopsies have revolutionized cancer diagnostics by enabling the detection of circulating tumor DNA (ctDNA), circulating tumor cells, and extracellular vesicles from peripheral blood samples. This minimally invasive approach allows for longitudinal monitoring of tumor evolution, clonal dynamics, and emerging resistance mutations. However, detecting low-frequency variants in plasma DNA is technically challenging due to background noise and sequencing errors, and the technology is far from mature. AI-based analytical frameworks improve sensitivity and specificity by distinguishing true tumor-derived mutations from technical artifacts. DL models trained on longitudinal ctDNA data can predict disease progression before radiological evidence becomes apparent. Furthermore, AI algorithms can integrate liquid biopsy results with imaging findings and clinical biomarkers to create dynamic predictive models. These approaches support adaptive therapy adjustments, optimize immunotherapy timing, and identify minimal residual disease after surgery or systemic treatments. In early cancer detection, AI-enhanced multi-cancer early detection procedures analyze cfDNA methylation patterns and cfDNA fragments to define the presence and tissue of origin of tumors, potentially enabling screening strategies that transcend organ-specific paradigms (1,5,9).

## AI-mediated target identification for novel disease therapies

Vast amounts of population-based data are now available that include WES and sometimes WGS combined with lifestyle information, geographical data, and prospective analyses of disease incidence. AI-mediated quantification of the correlation between genetic variants and disease incidence has the potential to identify not only disease-causing but also protective variants. The potential of such analyses cannot be overestimated because even variants with small effect sizes can provide unexpected insight into disease pathways and pinpoint genes not traditionally associated with a disease that play key deleterious or protective roles. This approach is particularly useful for chronic, relatively slowly developing diseases, such as neurodegenerative or neuropsychiatric disorders. For these diseases, clinical trials are cumbersome and expensive, and no effective disease-modifying therapies are available. For example, a cluster of rare variants in a gene not previously linked to Parkinson slightly increases disease risk when combined with high exposure to urban air pollution. Simply speaking, each variant alone has a tiny effect, but the AI recognizes their combined influence (10,11). Another example is related to a protective variant in a mitochondrial gene, which appears more frequently in individuals who remain disease-free into old age, even though they carry known risk alleles (10,12). Traditional analysis had overlooked this variant because its effect size is small and only becomes apparent when lifestyle factors are included.

## AI-driven translation of pharmacogenomics and nutrigenomics

Pharmacogenomics (PGx) investigates how genetic variation influences drug metabolism, efficacy, and toxicity. Genetic polymorphisms in drug-metabolizing enzymes, particularly cytochrome P450 (CYP) isoenzymes, and in drug transporters and pharmacodynamic targets are well-established determinants of interindividual variability in therapeutic response.

Variants in CYP genes – many of which are highly polymorphic across global populations – can markedly alter a drug’s metabolism and thereby influence both therapeutic outcomes and adverse-event risk. While single-gene PGx testing has entered clinical practice, comprehensive interpretation remains complex. AI-enabled decision-support systems increasingly address this complexity by integrating genomic information with clinical parameters such as renal function, age, comorbidities, and potential drug-drug interactions to improve the individualized selection and dosing of a drug. These systems generate personalized dosing recommendations and alternative drug suggestions, thereby minimizing adverse drug reactions and improving therapeutic outcomes. In oncology, AI-guided PGx analysis can optimize chemotherapy dosing and reduce toxicity. In cardiology and psychiatry, AI systems help tailor anticoagulant, antidepressant, and antiplatelet therapies based on genetic risk profiles. The integration of PGx into electronic health records, supported by AI algorithms, ensures that genomic information becomes actionable at the point of care (13,14).

Nutrigenomics explores how genetic variation influences metabolic responses to dietary components. Individual differences in lipid metabolism, glucose regulation, inflammation, and microbiome composition contribute to heterogeneous dietary responses. AI models that integrate genomic variants, metabolomic profiles, microbiome data, and continuous physiological monitoring can generate personalized nutrition plans. By analyzing dynamic glucose curves, lipid profiles, and inflammatory markers, AI systems adjust dietary recommendations in real time. Such precision nutrition strategies have implications for obesity management, metabolic syndrome prevention, and cardiometabolic risk reduction. AI-enabled nutrigenomic platforms synthesize host genetics with multi-omics and clinical context – eg, microbiome composition, metabolomic readouts, diet logs, and wearable data – to stratify who is more likely to benefit from targeted micronutrient supplementation or defined dietary patterns, thereby supporting risk-tailored prevention at scale.

For example, common functional variants in *BCMO1* can reduce conversion of β-carotene to vitamin A, implying that some individuals may require preformed retinol rather than provitamin A for optimal nutrition. Such genotype-nutrient relationships are the type of features AI models can operationalize when prioritizing interventions. At the level of fatty-acid metabolism, genetic variation in *FADS1/2* modulates the levels of long-term fatty acids and of polyunsaturated fatty acids status, thereby determining the response to omega-3 intake or supplementation – patterns that machine-learning frameworks can capture to recommend responders and avoid non-beneficial use. Beyond single nutrients, ML pipelines that integrate genomic, microbiome, clinical, and behavioral features already predict post-prandial glycemic responses and guide short-term personalized diets in randomized settings, illustrating a path from individual prediction to population-level preventive nutrition when deployed via a digital health infrastructure. Complementary reviews outline how precision nutrition is moving from one-size-fits-all guidance toward AI-driven, multi-omic models for prevention while also emphasizing the need for rigorous validation, standards, and equitable implementation before widescale public-health adoption (15).

## AI-enabled environmental assessments in biomedical research

Alongside genomic, clinical, and lifestyle data, modern biomedical data sets increasingly integrate environmental exposure information – ranging from climate variables to chemical pollutants. AI systems are uniquely suited to analyze these large multidimensional data sets, revealing subtle interactions between environmental factors and genetic susceptibility that traditional methods often miss. AI models combining satellite-derived temperature data, genomic sequencing, and longitudinal kidney-function measurements can detect how chronic heat exposure interacts with genetic variation. These analyses reveal both risk-enhancing variants in heat-stress response pathways and protective variants in genes regulating mitochondrial efficiency. Such findings highlight how environmental stressors shape disease trajectories over decades (16). On the other hand, environmental exposure to pesticides and airborne pollutants has long been suspected to contribute to neurodegenerative disorders, but the underlying mechanisms are difficult to disentangle because exposures are variable, cumulative, and often interact with genetic background. AI-enabled analyses allow researchers to integrate high-resolution environmental maps with genomic and clinical data to uncover these relationships. Using geospatial pesticide-application databases, residential histories, and whole-genome sequencing, an AI model can estimate each participant’s cumulative exposure to specific agricultural chemicals. When these exposure metrics are combined with genetic data and long-term neurological outcomes, the model identifies patterns such as the following:

1. Individuals carrying rare variants in genes involved in xenobiotic metabolism show a modest but consistent increase in the risk for developing Parkinson-like motor symptoms when living near areas with intensive pesticide use (17).

2. A variant in a gene regulating neuronal antioxidant defenses appears to reduce vulnerability to pesticide-related neurotoxicity. Carriers of this variant maintain healthier motor-function trajectories despite similar exposure levels. This protective effect becomes apparent only when the model integrates genomic data with detailed pesticide-exposure histories and prospective clinical assessments (18).

**Figure 2:**
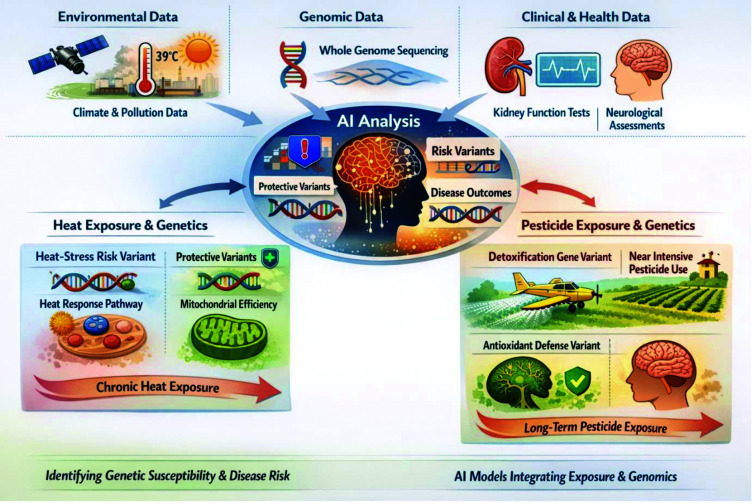
Synergistic processing of environmental data and genetic predispositions for clinically applicable health risk.

AI-enabled environmental analyses show that genetic susceptibility to pesticide exposure often arises from many variants, each of which exerts only a small influence on its own. AI algorithms can detect the combined influence of such variants when pesticide exposure is treated as a continuous, time-varying parameter. By integrating long-term exposure histories with genomic data, the model can identify individuals whose detoxification-pathway variants increase vulnerability to neurodegenerative outcomes (Figure 2).

## Ethical, infrastructural, and societal considerations

The integration of AI into medicine introduces a new class of ethical, regulatory, and infrastructural challenges that extend far beyond traditional clinical risk management. As health care systems become increasingly data-driven, the protection of genomic, phenotypic, and longitudinal health data becomes central to maintaining public trust. Recent analyses emphasize that vulnerabilities in digital health ecosystems –particularly those involving large-scale genomic repositories and AI-augmented clinical decision systems – demand robust encryption protocols, zero-trust cybersecurity architectures, secure cloud infrastructures, and continuous auditability to mitigate unauthorized access and algorithmic drift. The regulatory landscape remains fragmented. Many AI tools operate in a “liminal” space outside the clear jurisdiction of existing regulatory bodies, creating gaps in accountability, patient protection, and safety oversight. Even as agencies such as the Food and Drug Administration (FDA), European Medicines Agency, and Medicines and Healthcare products Regulatory Agency advance AI-specific pathways, unauthorized or insufficiently validated “shadow AI systems” continue to disseminate into clinical workflows, raising significant concerns about patient safety and equity. Algorithmic bias persists as a primary ethical threat. Systematic reviews show that many clinical AI models are trained on data sets that are demographically narrow, insufficiently diverse, or poorly representative of global populations. This leads to unequal model performance, exacerbated health disparities, and potential harm to historically underserved groups. AI models trained on homogeneous data sets may perform poorly in underrepresented populations, potentially exacerbating health care disparities. Equitable performance must be ensured by transparent model validation, explainable AI frameworks, and continuous post-implementation monitoring.

Furthermore, AI must remain an assistive tool rather than a replacement for clinical judgment. In this perspective, a physician’s role evolves from a sole decision-maker to an interpreter of AI-supported insights. Patients, empowered by predictive genomic information, become active participants in shared decision-making processes (1). Looking ahead, international guidelines such as the FUTURE-AI framework propose multi-stakeholder, globally harmonized infrastructures for trustworthy AI – emphasizing verifiable transparency, reproducibility, resilience, and sustainability. These next-generation frameworks will govern how AI systems are validated, audited, deployed, and continuously improved across borders, laying the foundation for a future in which AI becomes a safe, equitable, and indispensable clinical partner (19).

Another ethical consideration is access to information. As our world becomes increasingly fragmented and ruled by power politics, especially poorer countries run the risk of losing the ability to retrieve and use important information for their health care and research communities. This risk, as articulated by the UN Secretary-General’s Scientific Advisory Board briefs on global data governance and equitable access to scientific resources, threatens to create winners and losers in the world’s population. It could potentially even apply to “middle” countries, such as those of the European Union or Australia and New Zealand, which do not have the same AI resources as the USA and China. The European Union’s AI Act is the first broad regulatory framework designed to govern the development and deployment of AI across sectors, including health care. It categorizes AI systems by risk level and places the strictest requirements on those used in clinical decision-making. These requirements include transparency about training data, clear documentation of model performance, mechanisms for human oversight, and continuous monitoring once the system is in use. While the Act aims to ensure safety and accountability, it also raises concerns that smaller countries or institutions may struggle to meet the technical and administrative demands, potentially widening the gap between regions with strong AI infrastructure and those without it.

## Limitation of current AI systems

Although AI has made substantial progress in biomedical applications, several fundamental limitations remain. Many clinical AI models rely on large, well-annotated data sets; yet, real-world medical data are often fragmented, inconsistently recorded, or biased toward specific demographic groups (20). This mismatch can lead to a strong performance during model development but reduced reliability when systems are deployed in new clinical environments or applied to underrepresented populations.

A further challenge is the limited interpretability of many modern AI architectures, particularly deep learning models, which can make it difficult for clinicians to understand the basis of a prediction or to identify when a model is likely to fail. AI systems can also be sensitive to small variations in input data, such as differences in imaging protocols, laboratory measurement standards, or EHR documentation practices. Additionally, most models struggle with rare diseases or atypical presentations because these cases are insufficiently represented in training data sets. These limitations underscore the need for rigorous external validation, continuous performance monitoring, and careful integration into clinical workflows to ensure safe and equitable use.

## Future directions

The next wave of biomedical AI will likely be driven by systems capable of integrating multi-omics data – genomic, transcriptomic, proteomic, glycomic, metabolomic, epigenomic, and other profiles – together with imaging, clinical narratives, environmental exposures, and continuous physiological measurements from wearable devices. Merging these heterogeneous data streams into unified analytical frameworks will enable far more accurate modeling of disease biology and patient-specific risk. Such multimodal architectures are expected to support the development of digital twins: computational representations of individual patients that simulate disease progression, therapeutic response, and long-term outcomes under different intervention scenarios.

Advances in privacy-preserving computation, including federated learning, encrypted model training, and secure multiparty analytics, will allow international collaboration without requiring raw data to leave local institutions – an essential step for equitable global AI development. At the same time, regulatory frameworks such as the EU AI Act and the US regulatory initiatives on medical AI – including the FDA’s evolving framework for AI/ML-based medical devices and the US Executive Order on Safe, Secure, and Trustworthy AI – will shape how clinical AI systems are validated, monitored, and deployed, emphasizing transparency, robustness, human oversight, and continuous post-market performance evaluation.

In the preclinical research arena, the most important challenge is to move from AI-driven analyses of individual data-rich experimental results, such as WGS, proteomics, or transcriptomics, to an AI-driven integration of such data sets. Simply identifying pathways and overlaps in results is insufficient. The functions of most proteins and genes are poorly understood, and AI has up to now, even with the advent of AlphaFold, not been very helpful in assigning and validating protein and gene functions. Key, thus, will be to link AI-based hypotheses to actual experiments, which is a challenge in today’s world that expects rapid insights from AI instead of accepting the need for extensive AI-motivated experiments.

Looking ahead, the impact of AI in medicine and biomedical research will depend on broad access to high-quality multi-omics and clinical data sets, governance structures that ensure fairness and accountability, and sustained collaboration across scientific, clinical, and policy communities. These elements will determine whether AI becomes a universally accessible tool for improving health outcomes or a technology that deepens existing disparities.

## Conclusion

Machine learning builds computational models that detect patterns in complex data sets like genomic profiles, imaging studies, and electronic health records to produce clinically meaningful predictions. DL, a subset of ML, uses layered artificial neural networks loosely inspired by biological neurons to capture highly nonlinear features, making it especially powerful in image-dense fields such as radiology, pathology, and cardiology. More broadly, AI is becoming the integrative layer of modern medicine, unifying multi-omic, imaging, and clinical signals into decision-ready insights – accelerating whole-genome sequencing interpretation, improving molecular tumor profiling and liquid-biopsy analytics, and supporting individualized pharmacogenomic and nutrigenomic care.

The real promise of AI lies in augmenting rather than replacing human expertise, but realizing it requires honesty about current limits: narrow or unrepresentative data sets, weak external validation, uneven data quality (strong for standardized methods like WGS, far weaker for functional gene annotation or health records), and the risks of ungoverned “shadow AI” entering workflows. Responsible deployment demands prospective evaluation, post-deployment surveillance for algorithmic drift, bias testing, risk-appropriate explainability, and interdisciplinary governance. As the field bends toward multimodal, continuously learning models and patient-specific digital twins, these safeguards will determine whether AI meaningfully extends medicine from precision diagnosis to anticipatory, adaptive care for all patients.
